# Urinary Incontinence and Its Relationship with Obstetric, Age, and Ethnic Factors: A Cross-Sectional Study

**DOI:** 10.3390/healthcare13243254

**Published:** 2025-12-12

**Authors:** Cristian Santiago Torres, Katherine Geovanna Esparza, Verónica Alexandra Celi

**Affiliations:** Facultad Ciencias de la Salud, Universidad Técnica del Norte, Ibarra 100150, Ecuador; kgesparza@utn.edu.ec (K.G.E.); vaceli@utn.edu.ec (V.A.C.)

**Keywords:** urinary incontinence, obstetric factors, ethnicity, peoples, nationalities

## Abstract

Background/Objectives: To relate types of Urinary Incontinence (UI) with obstetric, age, and ethnic factors of interest—a cross-sectional study Methods, in the northern provinces of Ecuador. Methods: A descriptive, cross-sectional study was conducted with a study population of 2039 women with urinary incontinence (UI), recruited between October and November 2022 across different areas of the provinces of Imbabura, Carchi, and Esmeraldas. Data were collected using a characterization questionnaire and the International Consultation on Incontinence Questionnaire–Short Form (ICIQ-IU-SF). The data were analyzed using descriptive and inferential statistics. To quantify the association between risk factors and urinary incontinence, *p*-values and Odds Ratios (ORs) with 95% confidence intervals (CIs) were calculated. Results: A significant association with higher risk was observed between stress urinary incontinence (SUI) and women with a history of cesarean section (13.1%; OR = 1.34; *p* = 0.021). Likewise, SUI was more frequent among nulliparous women (22.6%; OR = 66.2; *p* < 0.001), young adults (34.4%; OR = 4.45; *p* < 0.001), and those of Karanki ethnicity (7.5%; OR = 2.74; *p* < 0.001). In contrast, urge urinary incontinence (UUI) was associated with vaginal delivery (93.9%; OR = 1.41; *p* < 0.001), multiparity (75.5%; OR = 1.78; *p* < 0.001), older age (41%; OR = 2.50; *p* < 0.001), and Awá ethnicity (12.7%; OR = 1.41; *p* = 0.009). Finally, mixed urinary incontinence (MUI) showed strong associations with cesarean section (21.8%; OR = 2.09; *p* < 0.001), grand multiparity (41.3%; OR = 4.54; *p* < 0.001), advanced age (31.1%; OR = 188.1; *p* < 0.001), and white women (24%; OR = 2.30; *p* < 0.001). Conclusions: Urinary incontinence in women is statistically associated with obstetric, age-related, and ethnic risk factors. These findings contribute scientific evidence specific to the Ecuadorian population, supporting the development of prevention and health promotion programs, as well as early interventions aimed at reducing the impact on women’s quality of life, health, and the economic well-being of their families.

## 1. Introduction

According to the World Health Organization (WHO), urinary incontinence (UI) is defined as the involuntary loss of urine that constitutes a hygienic or social problem and can be objectively demonstrated [1]. The global prevalence of UI in women ranges from 13% to 38.7%. In middle-aged women, prevalence reaches 30–40% and exceeds 50% in older women, making it the main symptom of genitourinary syndrome of menopause, affecting half of postmenopausal women [2].

UI represents a substantial economic burden for health systems, patients, and their families. Direct costs range from USD 153.71 to USD 32,709 per person, while indirect costs include an average of 160 h per year lost to presenteeism [3]. In the United States alone, the direct cost of UI care is estimated at USD 19.5 billion annually [4].

Spending on UI-related services for insured adult women decreased from USD 1401 in 2004 to USD 932 in 2013, with notable differences by race/ethnicity and insurance type: White and Hispanic women generally incur higher expenses than Black and Asian women [5]. For women over 65, annual costs per person can exceed USD 3500 [6].

UI is also a frequent complication after childbirth, and the risk varies by delivery type. Vaginal delivery nearly doubles the long-term risk of stress urinary incontinence, with an absolute increase of 8%, especially in younger women [7]. Forceps-assisted delivery is associated with an even higher long-term risk compared to other vaginal births in women under 50 [8]. Middle-aged women with a history of vaginal delivery show a significantly higher prevalence of all UI types compared to nulliparous women or those who delivered by other methods [9]. Both pregnancy and vaginal delivery increase UI risk in women aged 40–64, while cesarean section has a significant protective effect [10].

The relationship between parity and UI has been extensively studied, particularly in postpartum women. Evidence suggests a significant association between the number of children and UI severity, with higher parity linked to more severe symptoms [11].

UI becomes increasingly common with age. Among older women in low- and middle-income countries, prevalence varies widely but is highest in women aged 70 and above, underscoring the need for greater awareness, healthcare access, and culturally tailored interventions [12].

Prevalence and subtype distribution also differ by ethnicity in both women and men. Despite these variations, the distress caused by UI is consistently high across ethnic groups, with disparities in access to and type of treatment further influenced by ethnicity. Black and Asian American women have a lower risk of stress UI compared with White women, whereas Hispanic women show the highest overall prevalence. Urge incontinence, however, does not vary significantly by ethnicity after adjusting for risk factors [13,14,15].

A review conducted in Brazil by Lígia da Silva Leroy reported that stress UI was more common among White women, while urge UI was more frequent in Black women. Moreover, White and Asian women experienced less urine loss than Black and Hispanic women. This highlights the importance of examining UI prevalence by ethnicity worldwide [16].

Therefore, the aim of this study is to analyze the relationship between UI subtypes and the variables of delivery type, parity, age, and ethnicity. Female urinary incontinence shows significant associations with obstetric, age-related, and ethnic factors.

## 2. Materials and Methods

### 2.1. Study Design

A descriptive, cross-sectional study was conducted to characterize the different types of urinary incontinence in community health centers, their frequency, and their association with the proposed variables—namely obstetric, age, and ethnic factors—without establishing causality or evaluating temporal changes or incidence.

### 2.2. Setting

The women who participated in the study were located in different neighborhoods belonging to Ibarra, Caranqui, Natabuela, Cotacachi, and San Lorenzo, which correspond to the provinces of Imbabura, Carchi, and Esmeraldas, Ecuador.

### 2.3. Participants

The study population comprised 2039 women aged over 18. Participant recruitment was conducted using a non-probabilistic, convenience sampling method, in accordance with the pre-established selection criteria. This effort was part of a collaborative agreement between Universidad Técnica del Norte and the Ecuadorian Ministry of Public Health (MSP).

Potential participants were approached at general medicine outpatient clinics across various health sub-centers in the provinces of Esmeraldas, Carchi, and Imbabura. Each woman who agreed to take part completed two instruments: the validated “ICIQ-IU-SF” questionnaire, to identify the presence and type of urinary incontinence, and a characterization questionnaire capturing key sociodemographic and obstetric variables (age, type of delivery, number of children, and ethnicity).

As the study aimed to include all eligible women who consented to participate, no sample size calculation was performed. Instead, the research was carried out using the complete census of cases gathered throughout the study period.

### 2.4. Feasibility and Data Collection

The study was feasible as validated instruments were available to ensure accurate data collection. To assess the types of urinary incontinence, the ICIQ-IU-SF (International Consultation on Incontinence Questionnaire—Urinary Incontinence Short Form), was used in Spanish, which consists of three scored items (frequency, amount, and impact), was used along with a questionnaire for rapid and easy self-diagnosis that evaluates symptoms and the impact of urinary incontinence [17]. The validation of the Spanish version of the ICIQ-IU-SF shows adequate content validity, internal consistency, measured with Cronbach’s alpha, of 0.87, considered optimal, and intra-observer reliability, measured with the weighted Kappa index for items three, four and five, was 0.84, 0.86 and 0.80, respectively, also considered very good [18]. Participants also completed a general data form, which consisted of three items related to demographic information, family situation, and obstetric history for characterization purposes.

### 2.5. Data Analysis

Data processing was performed using Jamovi software 2.3.28 [19] designed for accessible and reproducible statistical analysis. Tests were applied to analyze the relationship between UI and obstetric, age, and ethnic factors, such as Pearson’s chi-square (χ^2^) test, which allowed for assessment of associations or differences between categorical variables [20] and the association between risk factors and urinary incontinence was quantified by calculating odds ratios (OR) and 95% confidence intervals (CI) [21]. Significance was set at *p* < 0.05.

## 3. Results

In Table 1, when characterizing the women, included in the study, the majority were found to be adults (43.5%), predominantly of mestizo ethnicity (58.8%). Most had a history of multiparity (53%) and gave birth through vaginal delivery (75.3%).

Regarding the results in Table 2 on the relationship between urinary incontinence and delivery mode, number of children, age, and ethnicity, the findings show that nulliparous women are at higher risk of developing stress urinary incontinence (OR = 66.178; *p* < 0.001), whereas vaginal delivery appears to exert a protective effect (OR = 0.716; 95% CI: 0.682–0.751). With respect to parity, having one child (OR = 66.178) or none (OR = 10.23) is associated with an increased risk of stress incontinence, while high multiparity seems to be linked to a reduced risk (OR = 0.06). In terms of age, younger women present a higher risk (OR = 4.446), whereas older women show a very low risk (OR = 0.021). Ethnicity also appears to play a role: women from the Karanki (OR = 2.747) and Afro-descendant (OR = 1.609) groups have a greater likelihood of experiencing stress incontinence. However, in other ethnic groups, such as Awá (OR = 0.985, *p* = 0.91, 95% CI: 0.759–1.278) and White (OR = 0.857, *p* = 0.202, 95% CI: 0.677–1.086), the associations were not statistically significant.

In Table 3, vaginal delivery is associated with a higher risk of urge urinary incontinence (OR = 1.41), while both cesarean section (OR = 0.39) and nulliparity (OR = 0.024) appear to have a protective effect. There is a clear relationship between the number of children and the risk of urge incontinence: for grand multiparous, the greater the risk (OR = 2.761); nulliparous (OR = 0.024) and primiparous women (0.055) demonstrate significant protection against this condition. Regarding age, intermediate older adult (OR = 2.749) are at greater risk for urge incontinence, whereas young adult (OR = 0.248) have the lowest risk. It is noteworthy that the advanced older adult group (OR = 0.029) appears to present significant protection; however, this result may be influenced by the small sample size (n = 1), limiting its statistical validity. In terms of ethnicity, women of Awa (OR = 1.418) and Mestiza (OR = 1.096) backgrounds present a slightly higher risk of urge incontinence. Conversely, the Karankis (or = 0.584), Afro-descendant (OR = 0.674), and White (OR = 0.694) groups show a protective effect. For the Natabuelas ethnicity, no statistically significant evidence was found (*p* = 0.247).

Table 4 shows that cesarean section is associated with a twofold increased risk of developing mixed urinary incontinence (OR = 2.09), while vaginal delivery does not show a statistically significant association (*p* = 0.286). Being grand multiparous significantly increases the risk of MUI, with a probability 4.5 times higher. In contrast, primiparous women demonstrate a protective effect against this condition (OR = 0.368). The advanced older adult (OR = 188.119) and intermediate older adult (OR = 6.212) age groups present the highest levels of risk for mixed incontinence, whereas young adult (OR = 0.342) show a lower probability of developing this pathology. From an ethnic perspective, women of White (OR = 2.303) and Mestizo (OR = 1.135) ethnicities present a higher risk of MUI. Conversely, the Awá ethnicity (OR = 0.282) shows significant protection. In the Afro-descendant group (*p* = 0.131), no statistically significant differences were identified.

## 4. Discussion

A study published in 2022 by Ushma and colleagues concluded that more than 60% of adult women in the United States have some type of urinary incontinence. The factors most strongly associated with this condition were age over 70 years—differing from our results—and a history of vaginal delivery, which is similar to our findings [22], On the other hand, in a retrospective cohort study of 172 multiparous women, it was observed that 30.2% had a higher prevalence of stress urinary incontinence (SUI), which is consistent with our results [23]; Finally, in a sample of 15,003 women with some type of urinary incontinence, 68% were non-Hispanic White women, 12% were non-Hispanic Black women, 8% were Mexican-American, 5% were other Hispanic, and 7% were from other ethnic groups [24], which differs from our research.

Women who underwent cesarean section had a moderate risk of SUI compared to vaginal delivery and some degree of protection against UUI, which coincides with the systematic review conducted by Press JZ et al., showing that cesarean section reduced the risk of postpartum stress urinary incontinence from 16% to 9.8% in 6 cross-sectional studies and from 22% to 10% in 12 cohort studies [25]. Similarly, in the study published by López et al. in 2021, which evaluated eleven systematic reviews, six found that, compared to vaginal delivery, there is a significant reduction in the risk of urinary incontinence associated with cesarean section [26].

On the other hand, Arias Amador (2021) reported that vaginal delivery causes greater injury to pelvic soft tissues and pelvic floor denervation, which is associated with mixed urinary incontinence (MUI) and stress urinary incontinence (SUI), but not with urgency urinary incontinence (UUI) [27]. This finding contrasts with our results, since in our population vaginal delivery was linked to a higher risk of UUI, whereas SUI appeared to have a protective effect and MUI did not reach statistical significance.

According to our findings, nulliparous women show a markedly higher risk of SUI, whereas in the case of UUI, a protective effect was observed. In our study, nulliparous women showed a higher risk of developing stress urinary incontinence (SUI), whereas their risk of urgency urinary incontinence (UUI) was lower; Notably, no cases of mixed urinary incontinence (MUI) were observed among nulliparous women; this is consistent with the findings of Pang et al., who reported a prevalence of stress urinary incontinence (SUI) of 0.9% in nulliparous Chinese women, compared with 0.3% for urgency urinary incontinence (UUI) [28]. In multiparous and grand multiparous women, the risk of stress urinary incontinence (SUI) is very low, whereas the likelihood of urgency urinary incontinence (UUI) increases in both groups. However, mixed urinary incontinence (MUI) is markedly higher in grand multiparous women, but not in multiparous women. These findings, in turn, contrast with those of Alghamdi et al. (2021), in which grand multiparity was associated with a higher risk of SUI; nonetheless, regarding UUI, the odds ratio for grand multiparous women is comparable in both studies [29].

According to our statistical data, women over 75 years of age are at higher risk of developing mixed urinary incontinence (MUI), followed by urgency urinary incontinence (UUI), while the risk of stress urinary incontinence (SUI) is minimal. In contrast, younger adult women show a higher risk of SUI. These findings do not align with the nationally representative survey conducted by Nahar Q, which reported that in this age group, SUI was the most prevalent (8.3%), followed by MUI (5.5%) and UUI (2.1%) [30]. However, our results are consistent with the analysis by Komesu et al. (2016), which concluded that women aged 80–90 years exhibit a higher incidence of MUI [31]. Finally the Norwegian EPINCONT study mentions that the prevalence of UI increases with age, with the lowest prevalence (12%) in women under 30 years and the highest (40%) in women over 90 years [32]. Finally, in 2024 Kozhumam et al. identified that the overall prevalence of UI was 2.6% and increases descriptively with age: from 0.5% in women 40–49 years old to 6.6% in those over 70 [12].

Across different ethnic groups, Awá women were found to have a high risk of urgency urinary incontinence (UUI) and a strong protective effect against mixed urinary incontinence (MUI). In the Afro-descendant population, there was a higher risk of stress urinary incontinence (SUI) and a protective effect against both UUI and MUI. Among White women, the risk of developing MUI was more than double, while UUI showed a protective effect. Karanki women exhibited significant protection against UUI but a higher risk of SUI. The mestiza group demonstrated a mild risk of UUI and MUI, with a slight protective effect against SUI. Finally, the Natabuela group did not show any statistically significant association with either SUI or UUI. This finding contrasts with that of Akbar et al, 2021, who reported that, while the prevalence of urgency urinary incontinence (UUI) does not differ significantly across racial or ethnic groups, stress urinary incontinence (SUI) and mixed urinary incontinence (MUI) are significantly less prevalent in Black women compared to White women [33]; however, it is important to note that most of these studies, as highlighted by Joo Lee et al, 2024, are conducted in predominantly non-Hispanic White populations—which on average represent 76% of participants—compared with only 7.8% Hispanic women and 7% Black women. This underrepresentation may compromise external validity and limit the generalizability of the findings [34].

## 5. Limitations

The main limitations of this study include, first, the potential bias arising from underreporting or overreporting of symptoms due to the reliance on self-reported data. Second, several relevant variables known to influence urinary incontinence—such as body mass index, hormonal status, alcohol or tobacco use, and physical activity—were not included in the analysis. Finally, the study was conducted only in three provinces in northern Ecuador (Imbabura, Carchi, and Esmeraldas), which may limit the generalizability of the findings to the national level or to international contexts.

## 6. Strengths

Regarding the strengths of this study, the most notable is the large sample size, with more than two thousand women diagnosed with urinary incontinence, which enhances the robustness of the statistical analyses. In addition, the inclusion of women from different communities and healthcare settings allowed data collection in a real-world context, thereby increasing the external validity of the findings. The use of an internationally validated questionnaire further strengthens the reliability of the data and facilitates comparison with studies conducted in other countries. Finally, the inclusion of multiple ethnic groups provides a novel perspective and highlights factors that have rarely been examined in previous research.

## 7. Conclusions

This study highlights the multifactorial nature of urinary incontinence in women, demonstrating that demographic, obstetric, and ethnic factors all play significant roles. Our findings suggest that urinary incontinence, in its different types, is a health condition that affects women regardless of their age or ethnic group. It is also associated with the number of children and the type of delivery.

## Figures and Tables

**Table 1:** Characterization of the study subjects by Age, Ethnicity, Number of Children and Type of Delivery.

Age		
Age Range	Frequency	Percentage
Adult (18–44 years)	886	43.4%
Young adult (45–59 years)	467	22.9%
Middle-aged adult (60–64 years)	10	0.5%
Young older adult (65–74 years)	495	24.3%
Intermediate older adult (75–84 years)	108	5.3%
Advanced older adult (>85 years)	73	3.6%
Total	2039	100%
**Ethnicity**		
Mestiza	1199	58.8%
Awá	207	10.2%
Karankis	111	5.4%
Natabuelas	76	3.7%
Afro-descendant	203	10.0%
White	243	11.9%
Total	2039	100%
**Number of children**		
Nulliparous (No births)	265	13%
Primiparous (1 birth)	435	21.3%
Multiparous (2–4 births)	1081	53%
Grand Multiparous (5 or more births).	258	12.7%
Total	2039	100%
**Type of Delivery**		
No delivery	265	13%
Vaginal delivery	1536	75.3%
Cesarean	238	11.7%
Total	2039	100%

**Table 2:** Statistical Association Between Stress Urinary Incontinence and the Variables: Type of Delivery, Number of Children, Age, and Ethnicity.

Stress Urinary Incontinence								
		*n*	%	*X* ^2^	*p*-Value	OR	IC 95%	
							INF	SUP
Type of delivery	No delivery	262	22.6	218.84	0.001	66.178	21.278	205.821
	Vaginal delivery	746	64.3	175.865	0.001	0.716	0.682	0.751
	Cesarean	152	13.1	5.345	0.021	1.339	1.044	1.719
Number of children	Nulliparous	262	22.6	218.84	0.001	66.178	21.278	205.821
	Primiparous	405	34.9	295.675	0.001	10.23	7.735	14.668
	Multiparous	474	40.9	159.579	0.001	0.592	0.545	0.642
	Grand Multiparous	19	1.6	295.415	0.001	0.06	0.038	0.095
Age	Adult	557	48	22.817	0.001	1.283	1.156	1.424
	Young adult	399	34.4	201.294	0.001	4.446	3.491	5.662
	Middle-aged adult			13.262	0.001			
	Young older adult	202	17.4	68.94	0.001	0.522	0.447	0.611
	Intermediate older adult			150.497	0.001			
	Advanced older adult	22	0.2	90.522	0.001	0.021	0.005	0.087
Ethnicity	Awá	117	10.1	0.013	0.91	0.985	0.759	1.278
	Afro-descendant	138	11.9	11.305	0.001	1.609	1.214	2.132
	White	129	11.1	1.628	0.202	0.857	0.677	1.086
	Karankis	87	7.5	22.101	0.001	2.747	1.763	4.279
	Mestiza	642	55.3	13.286	0.001	0.873	0.813	0.939
	Natabuelas	47	4.1	0.789	0.374	1.228	0.78	1.935

**Table 3:** Statistical association between urge urinary incontinence and the variables: type of delivery, number of children, age, and ethnicity.

Urge Urinary Incontinence								
		*n*	%	*X* ^2^	*p*-Value	OR	IC 95%	
							INF	SUP
Type of delivery	No delivery	3	0.5	133.852	0.001	0.024	0.0008	0.075
	Vaginal delivery	614	93.9	178.335	0.001	1.41	1.352	1.471
	Cesarean	37	5.7	33.787	0.001	0.39	0.278	0.547
Number of children	Nulliparous	3	0.5	133.852	0.001	0.024	0.008	0.075
	Primiparous	11	1.7	221.565	0.001	0.055	0.03	0.099
	Multiparous	494	75.5	196.014	0.001	1.782	1.653	1.922
	Grand Multiparous	146	22.3	81.476	0.001	2.761	2.198	3.467
Age	Adult	265	40.5	3.37	0.066	0.904	0.81	1.008
	Young adult	49	7.5	129.501	0.001	0.248	0.187	0.329
	Middle-aged adult	10	1.5	21.282	0.001			
	Young older adult	268	41	146.104	0.001	2.5	2.151	2.906
	Intermediate older adult	61	9.3	31.181	0.001	2.749	1.901	3.975
	Advanced older adult	1	0.2	32.762	0.001	0.029	0.004	0.211
Ethnicity	Awá	83	12.7	6.805	0.009	1.418	1.091	1.842
	Afro-descendant	49	7.5	6.518	0.011	0.674	0.495	0.917
	White	60	9.2	6.903	0.009	0.694	0.527	0.915
	Karankis	24	3.7	5.887	0.015	0.584	0.375	0.909
	Mestiza	409	62.5	5.544	0.019	1.096	1.017	1.182
	Natabuelas	29	4.4	1.341	0.247	1.307	0.83	2.056

**Table 4:** Statistical association between mixed urinary incontinence and the variables: type of delivery, number of children, age, and ethnicity.

Mixed Urinary Incontinence								
		*n*	%	*X* ^2^	*p*-Value	OR	IC 95%	
							INF	SUP
Type of delivery	No delivery			37.779	0.001			
	Vaginal delivery	176	78.2	1.138	0.286	1.043	0.969	1.123
	Cesarean	49	21.8	25.05	0.001	2.09	1.577	2.771
Number of children	Nulliparous			37.779	0.001			
	Primiparous	19	8.4	25.037	0.001	0.368	0.238	0.571
	Multiparous	113	50.2	0.793	0.373	0.941	0.821	1.079
	Grand Multiparous	93	41.3	188.223	0.001	4.544	3.672	5.623
Age	Adult	64	28.4	23.184	0.001	0.628	0.507	0.777
	Young adult	19	8.4	29.943	0.001	0.342	0.221	0.53
	Middle-aged adult			1.246	0.264			
	Young older adult	25	11.1	23.846	0.001	0.429	0.294	0.626
	Intermediate older adult	47	20.9	122.576	0.001	6.212	4.359	8.853
	Advanced older adult	70	31.1	555.306	0.001	188.119	59.73	592.481
Ethnicity	Awá	7	3.1	13.745	0.001	0.282	0.135	0.592
	Afro-descendant	16	7.1	2.283	0.131	0.69	0.422	1.128
	White	54	24	35.171	0.001	2.303	1.76	3.014
	Karankis			14.561	0.001			
	Mestiza	148	65.8	5.078	0.024	1.135	1.025	1.257
	Natabuelas			9.792	0.002			

## Data Availability

Data are reported in the manuscript and at link https://zenodo.org/uploads/17041902. Accessed on 2 September 2025.

## Abbreviations

The following abbreviations have been used in this manuscript:

UI	Urinary Incontinence
UUI	Urge Urinary Incontinence
SUI	Stress Urinary Incontinence
MUI	Mixed Urinary Incontinence
ICIQ-IU-SF	International Consultation on Incontinence Questionnaire
